# Willingness to Work during Public Health Emergencies: A Systematic Literature Review

**DOI:** 10.3390/healthcare10081500

**Published:** 2022-08-09

**Authors:** Gonçalo Santinha, Teresa Forte, Ariana Gomes

**Affiliations:** 1 GOVCOPP, Department of Social, Political and Territorial, University of Aveiro, 3810-193 Aveiro, Portugal; 2 Department of Social, Political and Territorial Sciences, University of Aveiro, 3810-193 Aveiro, Portugal

**Keywords:** healthcare professionals, emergency, threat, willingness, abilities, health systems

## Abstract

The identification of the factors underlying the willingness or lack thereof to respond to public health emergencies is paramount to informing more capable health services. The interest in this topic appears renewed with each surge of threat, either referring to natural disasters, man-made violence, or epidemic and pandemics. However, there is no systematic approach to the research patterns and related main findings concerning individual and contextual determinants. The present article contributes to this theme through a systematic literature review of a sample of 150 articles published in the last 30 years on the subject of willingness and preparedness of health professionals to deal with public health threats. Our findings show that the research is mainly phenomena and contextual driven, responding to whichever emergency threat is more salient in a given period. Geographically, research on this topic is led by USA and China, mostly solely, while European countries invest in collaborations that are more international. Universities, including health institutes and schools, and researchers at hospitals conduct most of the research on the topic. The main research areas are medicine, psychology, and psychiatry. Pandemics, including COVID-19, influenza, and natural disasters, are the phenomena gauging more attention as opposed to terrorism events and biological accidents. The specific role of health professionals within the institution, their belief in ethical duties, preparation training, and concerns regarding infection of self and family are the main variables influencing the willingness and ability to report to work in public health emergencies.

## 1. Introduction

The worldwide impact of the COVID-19 pandemic fostered the debate and research on a number of issues that required urgent understanding to face the challenges ahead and mitigate the negative impact of the circumstances. These included, among many others, the effects of public health communication, the citizens’ attitudinal and behavioral dispositions to engage and act in accordance with the governmental measures, the general view and compliance with vaccination programs, teleworking provisions, and related issues concerning work-personal life balance and gendered expectations [1]

However, these topics appear peripheral when compared with the most fundamental ones related to the coping of health professionals and the resilience of healthcare services imminently threatened [2]. As common to other public health emergency crises, including pandemic outbreaks as the current one, previous epidemics, natural, chemical, and radio nuclear disasters man-made disasters [3], the demand and pressure are felt especially on emergency medical services (EMS) [4]. When the challenges are unsurmountable, the emergency nature of the healthcare provision, however, may extend to other care services as evidenced by contingency measures applied in hospital facilities during the COVID-19 pandemic (e.g., ventilation provision, infectious wings organization, prioritizing of COVID-19 infected patients, reallocation of human resources). Medical services provision in emergency settings is thus a politically important dimension of healthcare [5], and there have been consistent calls for developing healthcare facilities and staff emergency preparedness. The most systematic longitudinal contribution is from the World Health Organization, which makes regularly available up-to-date, high-quality materials to assist health professionals as well as policy-makers in the prevention and response to different types of emergencies (e.g., [6,7]). Overall, the recommendations advocate for the development of partnerships, coalitions, objective goals, and, most of all, the promotion of willingness and capacitation of health professionals to better deal with risk-based contexts [4]. It is worth highlighting that health care professions are not inherently risk-based as other professions, such as the military. Henceforth, the stimulation of a mental mindset and optimal arousal of health workers requires tailored training for these skills to kick in in the face of a threatening event. Not only the responsiveness but also the psychological resilience and coping with the exposure to an unusual level of pressure, urgency, threat, and danger to what add common barriers such as lack of resources and inherent fatigue and burnout [2]. The allocated resources and the training of responsive coping should contemplate the wide diversity of potential emergencies [8], the willingness of healthcare professionals to exercise their activity in such scenarios, and, last but not least, the follow-up of psychological adjustment to these abnormal higher levels of stress and anxiety that may heighten the probability of secondary trauma and depression [9].

From a policy viewpoint, the state of emergency responsiveness is an indicator of the financial or organizational nature of the health system. The main evaluation indicators employed concern the EMS per se and are usually processual ones prior to specific care processes and outcomes, such as response times for ambulances and waiting times for patients.

The need for more systematic measures applied is corroborated by the research showing the lack thereof. For example, [10] show that regular medical students are not prepared to deal with emergency situations, such as natural disasters consequences, as opposed to those who studied medicine in a military school. The same unpreparedness of health professionals to deal with emergencies, ranging from lack of knowledge of protocols and their implementation, was also found in Saudi Arabia, China, and Egypt [11,12].

Considering that poor planning or ineffective use of resources carries major negative consequences in emergencies [13], it is crucial to review emergency scenarios, locally and globally, and understand the factors underlying the suitability and preparedness of human resources. Some measures to improve doctors’ and nurses’ emergency skills include evaluations by physicians that are more senior or emergency medicine training as part of postgraduate practice [5].

Access to the risk perception and willingness to deal with emergency scenarios is beneficial for the health professionals, hospital administrators, and, in a certain way, political decision makers who may design and implement policies to manage these scenarios efficiently and with the necessary resources.

There is still a lack of an evidence-based universal set of performance indicators, and much remains unexplored in cross-country learning on best practices [5]. The present research intends to contribute to this gap with a systematic review of publications that address all kinds of public health emergencies, according to Prisma-P guidelines. The analysis is focused on bibliometric trends and research patterns on the type of public health emergency and health professional samples, as well as the variables found to affect the willingness to report to work during public health emergencies.

## 2. Materials and Methods

### 2.1. Approach

This study adopts a systematic literature review approach, following the PRISMA-P checklist, aimed to identify and analyze the literature on public health emergencies and health professionals’ willingness to report to work under those conditions. The analysis is guided by the following specific goals:

1. To identify the publication patterns and trends on this topic over time, including authorship, type of institutions, geographical contexts, publication venues ranking, and degree of internationalization and interdisciplinarity;
2. To explore which public health emergencies and health professional samples have been addressed over time as well as types of calamities and variables affecting willingness to work under emergency conditions;
3. To explore the main methodological patterns and themes over time and research fields.

### 2.2. Databases

The identification of the articles resulted from a search conducted between October and December 2021 on two databases, SCOPUS and PubMed, chosen due to their broadness in the field of medicine, social sciences, biomedicine, and health. Scopus has a broader coverage of academic journals (over 20,000 peer-reviewed ones) and is the most commonly employed scholar database to conduct systematic literature reviews [14]. Pubmed is the scientific platform of biomedicine areas more used in those specific fields, with 2.5 million users daily, 3 million searches, and 9 million pages [15]. It is open access and includes 33 million publications concerning life and health sciences, behavioral science, chemistry, and bioengineering [16].

### 2.3. Sample

The search for relevant literature on SCOPUS and PubMed included articles, conference papers, and book chapters on the fields of medicine, social sciences, health, biomedicine, and multidisciplinary published until 2021. On SCOPUS, the following Boolean codes were used: pandemic OR disaster* OR “public health emergencies” OR COVID*) AND (“willing* to work” OR “report* to work”). On PubMed, the Boolean codes were: (((((pandemic[Title/Abstract]) OR (disaster[Title/Abstract])) OR (public health emergencies[Title/Abstract])) OR (COVID-19[Title/Abstract])) AND (willing to work[Title/Abstract])) OR (report to work[Title/Abstract]).

No time period was applied, and only publications in English and Portuguese languages were retained, with the exclusion of French and German articles for which we did not have the required language skills to analyze. In addition, errata and letters were excluded.

**Figure 1:**
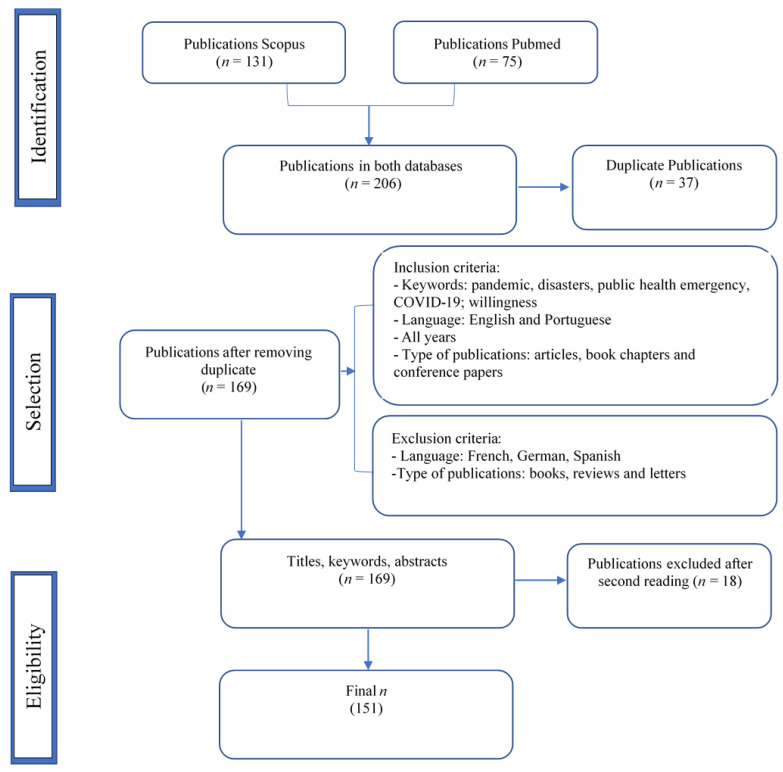
PRISMA flowchart.

Following these criteria, 206 publications were identified, 131 in the SCOPUS database and 75 in PUBMED, with 37 duplicates that were excluded, resulting in 169 publications. A second reading to determine the eligibility of the publications by titles, keywords and abstracts was made by two independent reviewers, and 18 publications were excluded due to focusing on tangential aspects of the topic under analysis. The final number of publications was 151 (as shown in Figure 1 describing the PRISMA flowchart screening processes for narrative synthesis).

### 2.4. Data Analysis

The final sample of 151 publications (148 focused on primary data and 4 on secondary data) selected from the two databases (SCOPUS and PubMed) were saved in bibtex and converted in R in bibliometrix, allowing the analysis of the bibliographic patterns of each publication. More specifically, it characterizes the geographic dissemination of this research as also the authors and institutions that may be specialized in the subject or its specific dimensions, answering to our first goal. A manual and software-based content analysis was conducted. First, the theoretical models, samples, and phenomena were identified manually to answer our second goal. In a second phase, a lexicographic analysis, hierarchical descending cluster analysis, and post-hoc correspondence analysis were conducted using the textual analysis software Iramuteq version 0.6. This software provides the users with different text analyses, either simple ones, such as the basic lexicography (e.g., word frequency), or multivariate ones, such as hierarchical descending analysis, herein used. The hierarchical descending cluster analysis is an iteration method that results in a hierarchy of clusters. The corpus was separated into 150 texts corresponding to the number of abstracts to be analyzed. Nouns and verbs were considered for analysis, as they were suitable to reflect emergent themes and theoretical and methodological aspects. The hierarchical descending cluster analysis retained 512 text segments and 2854 elementary context units (ECUs) from the total of (99.45%). The ECUs are text units within which IRAMUTEQ calculates the frequency of word co-occurrences. The aim of this analysis was to determine the more frequent themes of the research of public health emergencies and interrelated notions, thus meeting our third goal. The vocabulary distribution is presented in a comprehensive and clear way with graphical representations derived from the lexicographic analysis [17]. The main global information collected from the publications is systematized in Supplementary Table S1.

## 3. Results

### 3.1. Patterns by Year, Authorship, Countries of Origin, and Outreach Dissemination

#### 3.1.1. Evolution by Year

**Figure 2:**
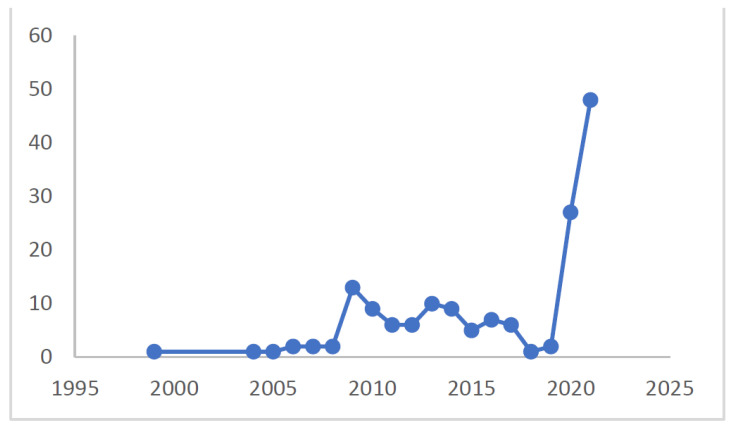
Evolution of publications by year.

The publications related to our search words, including pandemic, disasters, public health emergency, COVID-19, and willingness, as shown in Figure 2 below, are mostly concentrated in 2020 and 2021, probably fostered by the worldwide impact of COVID-19 and the attention drawn on the topic. Before, the publications were relatively absent until 2009, when there was a peak coincident with the influenza A pandemic, also called swine flu, which had the first recorded cases in Mexico before a global widespread. Until 2017, publications on the topic of emergency services and willingness were higher than before with some variation, especially in 2013, 2014, 2016, and 2017. These patterns suggest that the research on this topic is very much responsive to the threat at hand; hence, there may be a lack of perspective and follow up on different threats, including the emotional and psychological consequences afterward (e.g., PTSD) (e.g., [9,18]) or the long-term preparedness of systemic elements.

#### 3.1.2. Main Authors

**Figure 3:**
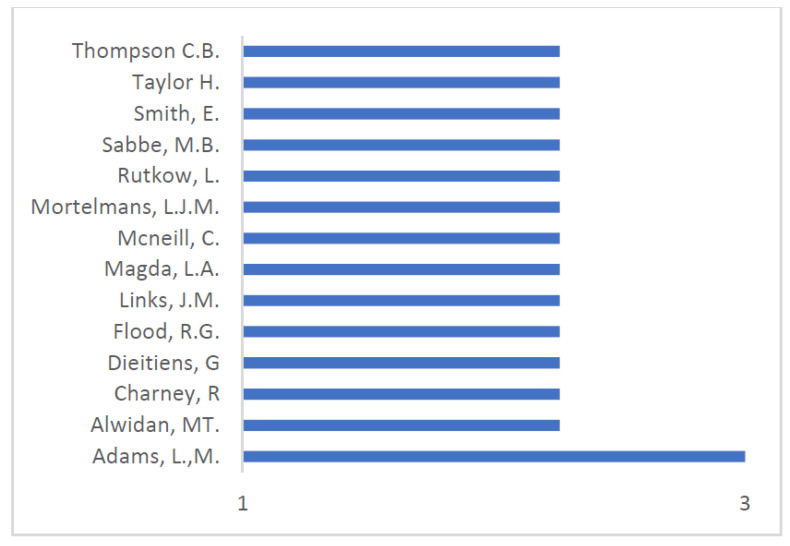
Ranking of main authors.

The author with more publications is from the USA and publishes in health sciences, albeit with only three articles (Figure 3; Table 1). The remaining authors have around two and one publications. The scarce contributions from each author suggest that this is not a topic of longitudinal research by any of them but rather, as indicated by the analysis by year, a response to specific crises.

#### 3.1.3. Countries of Origin of First Authors

**Figure 4:**
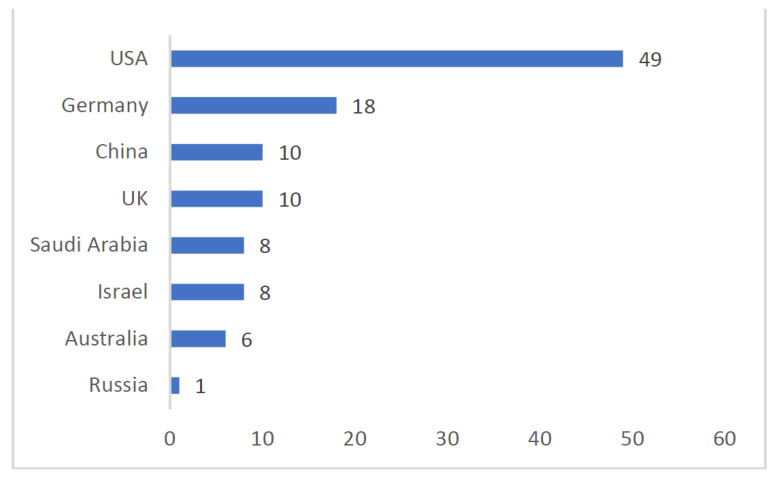
Ranking of countries of origin of first authors.

As shown in Figure 4 there is a wide diversity of countries of origin of the articles justified by the research focus on public health’s serious threats, such as epidemics and pandemics, and their large-scale impact on health systems and personnel. The majority of the contributions are, however, from a world powers context, such as the USA, Germany, China, and the UK, to what contributes that some of the emergencies, as opposed to pandemics, occurred specifically in those geographical settings, namely natural disasters and terrorist events. This higher research focus may also reflect a critical concern from the respective country’s institutions and researchers regarding these issues at local or national levels. What is more, these results indicate which contexts are still lacking empirical evidence on the willingness to work during public health emergencies.

#### 3.1.4. Articles’ Ranking per Citation

The number of articles’ citations is a suitable indicator of how widespread and disseminated the research is. The analysis was performed using Plum x Metrics, which allows us to see the usage and reads, captures, dissemination in social media, and mentions (Table 2).

The more cited articles focus on capacity needs for health care systems, factors that determine the ability and willingness to contribute during catastrophic events, and those that determine the lack thereof. A common topic among the more cited is ethical professionals’ duty in emergencies that may affect them visa vis the fear of contracting or transmitting to their families the infectious disease at hand. Interestingly, the one more disseminated in social media focuses precisely on perceived barriers to ability and barriers to willingness, concluding that there is an overlapping between both. The need to stimulate both abilities and willingness of health professionals [19] should consider the complex interaction between both, often mediated by feelings of fear and uncertainty toward risk.

**Table 2:** Ranking of articles per citation.

Article	Citations	Usage	Captures	Social Media	Mentions
[20]	267	129	144	1	6
[21]	112	1417	419	287	0
[22]	85	1336	291	0	0
[23]	80	12,452	178	0	0
[24]	79	2	79	0	
[25]	64	855	262	0	3
[26]	55	920	208	35	3
[27]	50	0	0	0	0
[28]	47	36	181	7	0

#### 3.1.5. Scientific Journal Ranking

The publication venues are very fragmented, with one journal comprising seven articles, the other three journals encompassing six, five, and four articles, respectively, and the remaining with three or fewer articles published in diversified journals. The more common is specific to disaster research (disaster medicine and public health preparedness; American Journal of Disaster Medicine), followed by generalist journals on public health (International Journal of Environmental Research and Public Health; Plos One Journal). Reinforcing the scarce publications per author, the wide dispersion of publication venues, with few journals publishing more than three articles on this subject, is also suggestive of the lack of a centralized corpus of research or specialized venue encompassing all the different emergency manifestations and valences. The more frequent disciplinary field is medicine (Table 3).

#### 3.1.6. Institutions of Authors, Degree of Internationalization and Interdisciplinarity

The authors’ affiliations are mostly linked to universities (*n* = 94) together with other educational institutions, namely health institutes (*n* = 4) and health schools (*n* = 6), followed by hospitals (*n* = 13) and specific medical departments (*n* = 10).

With the world being more interconnected than ever, it would be expected to notice the prevalence of global teams in international organizations rapidly rising and, accordingly, academic research becoming more international [29,30]. However, as shown in Table 4 below, most of the articles refer to studies conducted in only one country and by authors of solely one institution. Still, a significate number of articles show interactive collaborations among more than one institution and the involvement of multinational teams.

USA-based research shows a higher level of interinstitutional collaboration among different internal states. China is the country with fewer international collaborations as opposed to European countries that foster more international collaborations among them. These results are particularly understandable in the case of infectious diseases that may more easily expand to closer geographical contexts as common in European contexts.

The authors are mainly anchored in universities, mostly collaborating among them, while hospitals, in turn, tend to conduct research internally.

### 3.2. Methodological Patterns

#### 3.2.1. Sample and Object

As shown below in Table 5, doctors and nurses are the types of professionals and health care providers more addressed in this field. Students of the respective professions, particularly medicine, are next, followed by administrative/healthcare managers and pharmacists. The existence of different samples suggests that the research on the willingness and preparedness of health professionals is aware of the systemic contribution of different professions to more efficient emergency response.

One hundred and one articles are focused on hospital facilities. Twelve of them relate only to doctors and their availability to work under threatening circumstances, the need for individual protection and better team coordination to mitigate mental health issues. Thirty-one articles are exclusively focused on nurses with different focuses, namely their willingness to work after their schedules, the role of ethics and responsibility of care, and the willingness depending on personal responsibilities regarding children and relatives. Twenty-four articles are centered on doctors, nurses, and other health professionals.

Ten articles focus on primary health care facilities in articulation with other professions, including medical dentists and firefighters, both showing a willingness to collaborate in emergency settings. The tertiary healthcare, including medical expertise such as orthopedics or nursing home and palliative care workers, report not feeling skilled to assist patients in emergency contexts; however, they would be willing to do so.

#### 3.2.2. Type of Calamity

As shown in Table 6 below, the literature focuses on several types of specific calamities, with higher emphasis on infectious diseases, mostly with the recent COVID-19 pandemic (*n* = 53) and the pandemic influenza of 2009 and 2010.

The articles researching willingness and preparedness of health professionals during the COVID-19 pandemic correspond to a wide array of geographical contexts, given the worldwide reach of this threat: Palestine [32], Australia [33,105], Saudi Arabia [95], China [58,73,74,97,106], Ethiopia [37,107], Bangladesh [38], Spain [32], South Korea [43,47], Germany [46,108], Nepal [84,99], Qatar [82], Canada [42], Jordan [35,36], and Singapore [100].

Thirty-five other studies refer to other pandemics, particularly influenza virus-based that required special care in health units. These also include several geographical contexts: United States [24,52,91,109,110], Canada [111,112], China [51], Japan [113], Australia [23,114,115], England [21], and Germany [22].

As for natural disasters, ranging from higher to lesser impactful ones, they were conducted mainly in places where these disasters are more common, including Israel [70], Canada [75], United States [32,116], and Japan [79]. These studies, usually following disasters (e.g., earthquakes, floods), put a great emphasis on preparedness for future incidents.

Articles researching the coping of health professionals and the required measures in case of future attacks in the face of terrorist attacks are circumscribed to the U.S. [117,118].

**Table 6:** Type of calamity.

Type of Calamity	References
COVID-19	[28,31,32,33,35,37,38,42,43,45,46,47,48,58,60,74,81,82,83,84,85,95,97,99,100,101,105,106,107,108,119,122,123,124,125,126,127,128,129,130,131,132,133,134,145,146,147,148,149,150,151]
Infectious diseases	[39,96]
Natural disaster	[8,10,11,12,20,34,54,55,57,59,61,62,63,64,65,66,67,68,69,70,71,79,80,86,87,103,104,116,120,121,141,143,152,153]
Pandemic influenza	[21,22,23,24,25,26,49,50,51,52,53,91,92,94,111,113,114,115,135,136]
Pandemics (other type)	[40,76,109,110,137,138,139,140,142,144,145,154,155,156]
Terrorism event	[72,117,118]
Biological accident	[78,79,117,146,147,148,149,150,151,152,153,154,155,156,157,158,159]

#### 3.2.3. Instruments of Data Collection and Analysis

The main instruments used to collect data were questionnaires, interviews, and, to a lesser extent, focus groups. The questionnaire is employed more often (e.g., [34,46,47,70,72,76,95,96,99,114]) followed by interviews (e.g., [34,116]) and focus groups [21,71,100].

Regarding the data analysis patterns, besides descriptive statistics, the more commonly employed are linear and multiple regression analyses. In [95], the goals were to understand which factors contribute to the performance driven by ethical duty. Other studies employ the same analysis to examine the influence of parental stress and potential risks on nurses’ willingness to work in public health emergencies (e.g., [55,69]) or the evaluation of public emergencies and threats and the relation with the institutional response and work-related factors [43]. Logistic analyses were also conducted in [37,39,47,73,84,117]. In the studies of [32,64,69,109,117], chi-square tests were conducted in order to explore significant differences between willingness, attitude, and belief in their professional duties.

#### 3.2.4. Factors Affecting Willingness to Work in Emergency Situations and Contexts

The studies researching socio-demographic factors indicate that older health professionals show less willingness to work in emergency contexts, as do women and health professionals with more family responsibilities, especially small children and, also, pets to take care of (e.g., [8,63,80,142]). These results also reflect the gendered effects on the healthcare workforce [1].

Socio-demographic features and the type of emergency are thus fundamental variables impacting the willingness to report to work in public health emergencies. The radiological and nuclear incidents are the hazards associated with less willingness to report to work [96,146] also attributed to a lack of formal education and awareness of radiation-related events. The reported fear concerns more their own personal health, while for other infectious diseases, the fear is of contracting an infectious disease and spreading it to family members [6,9,37,80,82,89], especially to more vulnerable ones, such as children and the elderly.

Individual features of healthcare professionals, including fear, lack of confidence, and uncertainty about safety, are the more systematic variables predicting the willingness to report to work in several types of public health emergencies [37]. Other institutional-based variables also affect the willingness, namely the specific role and ability to perform the same function systematically in a chaotic context (e.g., [143,145]), as is to know exactly the responsibilities, duties, and functions of each professional [5], so as to counterbalance the threats with organization and efficiency.

On a supra level, tackling the same need to mitigate the uncertainty brought by a public health crisis is the need for awareness from health professionals to know the steps of the emergency protocol or plan, and tailored changes in the workplace to accommodate pandemic challenges (e.g., [143,147,148,149,150]). Another crucial factor is the symbolic commitment to their duties as caretakers [5,80,125].

##### Thematic Analysis through Hierarchical Descending Cluster Analysis

**Figure 5:**
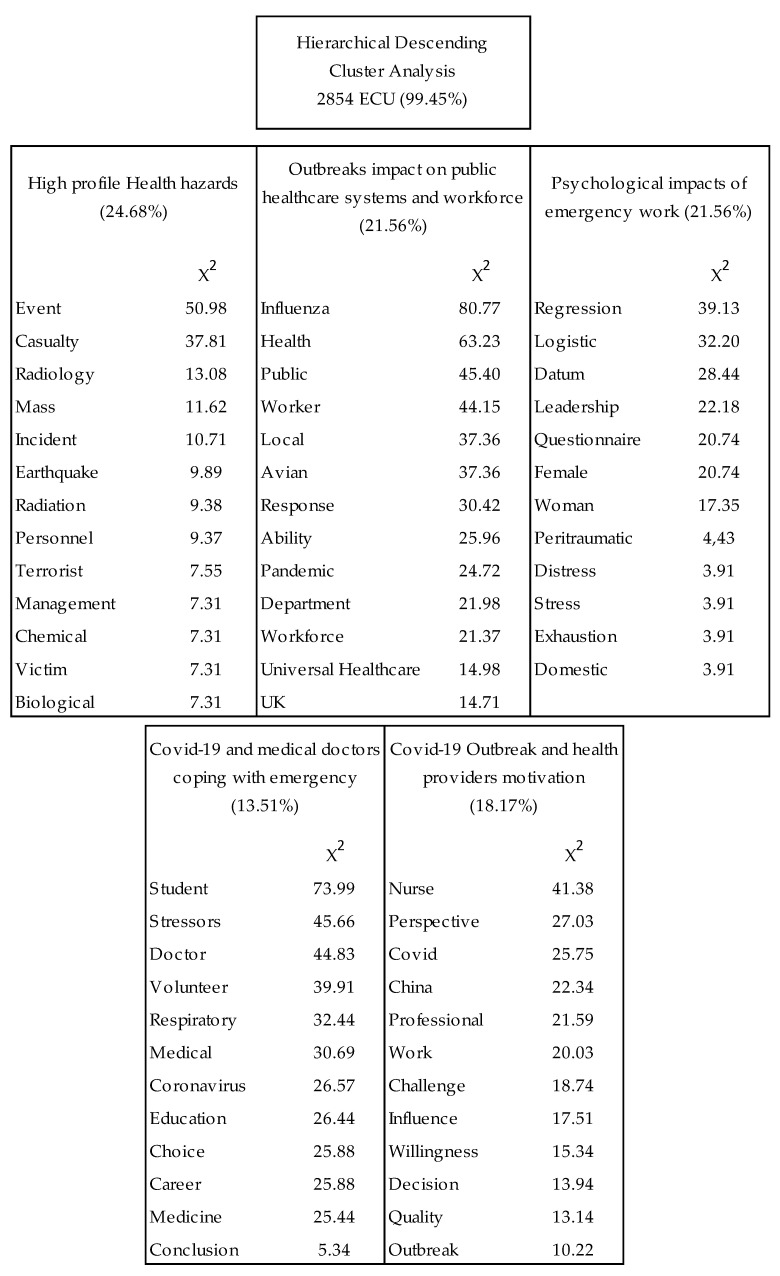
Five clusters extracted from hierarchical descending cluster analysis.

**Figure 6:**
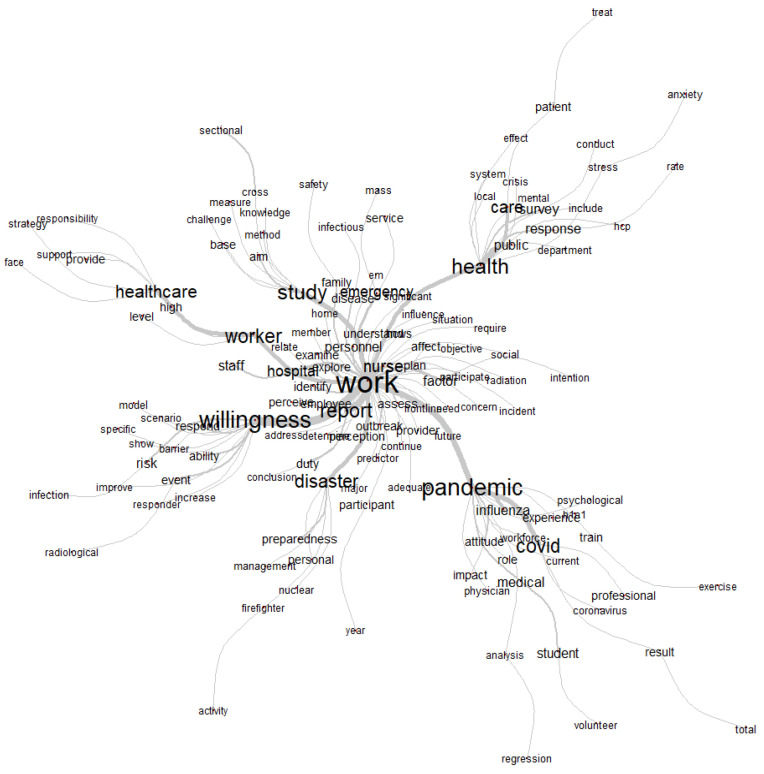
Most frequent expressions.

The hierarchical descending cluster analysis divided the corpus into 5 clusters distributed as shown in Figure 5 and Figure 6. Each cluster extracted through this method pertains to a specific theme, open to interpretation. The association strength between each word and its cluster is expressed through the χ² value; thus, the higher this value is, the more relevant the word for the cluster. As such, it allows an overview of thematic patterns of research and also of the elements that characterize each one (e.g., samples, specific topics, methodological approaches).

Cluster 1, labeled “High profile Health hazards”, accounts for 24.68% of our sample and focuses on a wide variety of public health risks and threats caused by natural events or man-made situations. The literature associated with this cluster emphasizes the need for strategic preparedness and readiness of healthcare workers and systems to resiliently deal with these dangerous hazards. It includes the following expressions: event (χ² = 50.98, *p* < 0.01), casualty (χ² = 37.81, *p* < 0.01), radiological (χ² = 13.08, *p* < 0.01), mass (χ² = 11.62, *p* < 0.01), incident (χ² = 10.71, *p* < 0.01), earthquake (χ² = 9.89, *p* < 0.01), nuclear (χ² = 4.91, *p* < 0.01), radiation (χ² = 9.38, *p* < 0.01), terrorist (χ² = 9.37, *p* < 0.01), personnel (χ² = 9.38, *p* < 0.01), management (χ² = 7.55, *p* < 0.01), chemical (χ² = 7.31, *p* < 0.01), victim (χ² = 7.31, *p* < 0.01), biological (χ² = 7.31, *p* < 0.01), guideline (χ² = 7.31, *p* < 0.01), family (χ² = 6.95, *p* < 0.01), terrorism (χ² = 6.7, *p* < 0.01), train (χ² = 6.17, *p* < 0.01), disaster (χ² = 5.26, *p* < 0.01), nonterrorism (χ² = 9.23, *p* < 0.01), smallpox (χ² = 9.23, *p* < 0.01), radioactive (χ² = 9.23, *p* < 0.01), oncology (χ² = 9.23, *p* < 0.01), accident (χ² = 9.23, *p* < 0.01), educational (χ² = 8.34, *p* < 0.01), bomb (χ² = 8.34, *p* < 0.01), preparedness (χ² = 6.92, *p* < 0.01), confidence (χ² = 5.78, *p* < 0.01 national (χ² = 5.44, *p* < 0.01), need (χ² = 5.01, *p* < 0.01), emergency (χ² = 4.89, *p* < 0.01), safety (χ² = 4.73, *p* < 0.0).

Cluster 2, named “Outbreaks impact on public healthcare system and workforce” (21.56% of our sample), focuses on other diseases that require emergence responses from the healthcare system and workers, namely influenza and H1n1 (avian flu). The articles associated with this cluster (accounting for 21.56% of the total) emphasize more healthcare public policy provisions alongside specific challenges faced by the healthcare workforce, such as absenteeism. It includes the words influenza (χ² = 80.77, *p* < 0.01), health (χ² = 63.23, *p* < 0.01), public (χ² = 45.4, *p* < 0.01), worker (χ² = 44.15, *p* < 0.01), local (χ² = 37.36, *p* < 0.01), avian (χ² = 37.36, *p* < 0.01), response (χ² = 30.42, *p* < 0.01), ability (χ² = 25.96, *p* < 0.01), pandemic (χ² = 24.72, *p* < 0.01), department (χ² = 21.98, *p* < 0.01), workforce (χ² = 21.37, *p* < 0.01), universal healthcare (χ² = 14.98, *p* < 0.01), U.K. (χ² = 14.71, *p* < 0.01), surge (χ² = 14.25, *p* < 0.01), ethical (χ² = 13.8, *p* < 0.01), state (χ² = 13.75, *p* < 0.01), system (χ² = 12.91, *p* < 0.01), potential (χ² = 11.09, *p* < 0.01), catastrophic (χ² = 11.0, *p* < 0.01), ill (χ² = 11, *p* < 0.01), emergency (χ² = 10.82, *p* < 0.01), global (χ² = 10.49, *p* < 0.01), absenteeism (χ² = 10.23, *p* < 0.01), capacity (χ² = 10.23, *p* < 0.01), unit (χ² = 10.23, *p* < 0.01), ethic (χ² = 10.23, *p* < 0.01), facility (χ² = 7.33, *p* < 0.01), duty (χ² = 6.84, *p* < 0.01), government (χ² = 6.83, *p* < 0.01), behavior (χ² = 6.83, *p* < 0.01), plan (χ² = 10.49, *p* < 0.01) flu (χ² = 5.34, *p* < 0.01), employee (χ² = 4.86, *p* < 0.01), numb (χ² = 4.43, *p* < 0.01), clinician (χ² = 4.43, *p* < 0.01), h1n1 (χ² = 3.91, *p* < 0.01), threat (χ² = 3.91, *p* < 0.01).

Cluster 3, named “Psychological impacts of emergency work”, accounts for 21.56% of the articles and includes more information on specific statistical analysis and instruments of data collection, but, more importantly, analyzes the psychological consequences of working in emergency situations. These include an allusion to emotional and socially demanding situations for mental health emergencies, including distress, stress, anxiety, peritrauma, and exhaustion, felt particularly by female participants. The concepts more associated with this cluster are regression (χ² = 39.13, *p* < 0.01), logistic (χ² = 32.2, *p* < 0.01), datum (χ² = 28.44, *p* < 0.01), leadership (χ² = 22.18, *p* < 0.01), questionnaire (χ² = 20.74, *p* < 0.01) female (χ² = 20.74, *p* < 0.01) woman (χ² = 17.35, *p* < 0.01), peritraumatic (χ² = 4.43, *p* < 0.01), distress (χ² = 4.43, *p* < 0.01), stress (χ² = 3.91, *p* < 0.01), exhaustion (χ² = 3.91, *p* < 0.01), domestic (χ² = 3.91, *p* < 0.01), proportion (χ² = 3.91, *p* < 0.01), disorder (χ² = 3.91, *p* < 0.01), support (χ² = 10.88, *p* < 0.01), collaboration (χ² = 10.23, *p* < 0.01), anxiety (χ² = 9.32, *p* < 0.01), status (χ² = 7.33, *p* < 0.01), intervention (χ² = 6.83, *p* < 0.01), emotional (χ² = 6.83, *p* < 0.01), social (χ² = 4.43, *p* < 0.01), scale (χ² = 5.34, *p* < 0.01), interview (χ² = 5.34, *p* < 0.01), mental (χ² = 4.91, *p* < 0.01).

Cluster 4, named “COVID-19 and medical doctors coping”, includes articles closer to those of cluster 1 also revolving around COVID-19, albeit with a broader range of issues and specific healthcare professions. It accounts for 13.51% of the articles. The emphasis is on students of medicine and medical doctors coping with pandemic COVID-19 stressors in situ and the application of educational and scientific-based approaches to deal with the new challenging outbreak. While Cluster 1 emphasized the burden on nurses, this cluster explores more in depth the psychological impact on doctors. The stronger words associated are student (χ² = 73.99, *p* < 0.01), stressors (χ² = 45.66, *p* < 0.01), doctor (χ² = 44.83, *p* < 0.01), volunteer (χ² = 39.91, *p* < 0.01), respiratory (χ² = 32.44, *p* < 0.01), medical (χ² = 30.69, *p* < 0.01), coronavirus (χ² = 26.57, *p* < 0.01), education (χ² = 26.44, *p* < 0.01), choice (χ² = 25.88, *p* < 0.01), career (χ² = 25.88, *p* < 0.01), medicine (χ² = 25.44, *p* < 0.01), conclusion (χ² = 5.34, *p* < 0.01), infection (χ² = 21.31, *p* < 0.01), disease (χ² = 19.8, *p* < 0.01), specialty (χ² = 19.36, *p* < 0.01), military (χ² = 19.36, *p* < 0.01), cause (χ² = 19.36, *p* < 0.01), infectious (χ² = 16.32, *p* < 0.01), Chinese (χ² = 16.32, *p* < 0.01), burden (χ² = 14.74, *p* < 0.01), anxiety (χ² = 14.69 *p* < 0.01), syndrome (χ² = 13.08, *p* < 0.01), parent (χ² = 13.08, *p* < 0.01), symptom (χ² = 13.08, *p* < 0.01), educate (χ² = 13.08, *p* < 0.01), satisfaction (χ² = 13.08, *p* < 0.01), depression (χ² = 13.08, *p* < 0.01), decrease (χ² = 11.62, *p* < 0.01), psychological (χ² = 10.71, *p* < 0.01), efficacy (χ² = 9.89, *p* < 0.01), (χ² = 4.91, *p* < 0.01), COVID (χ² = 9.38, *p* < 0.01), cope (χ² = 9.37, *p* < 0.01), stress (χ² = 9.38, *p* < 0.01), fear (χ² = 7.55, *p* < 0.01), expose (χ² = 7.31, *p* < 0.01), worry (χ² = 7.31, *p* < 0.01), emerge science (χ² = 7.31, *p* < 0.01), depressive (χ² = 7.31, *p* < 0.01), exposure (χ² = 6.95, *p* < 0.01), frontline (χ² = 6.7, *p* < 0.01), mental (χ² = 6.17, *p* < 0.01), male (χ² = 5.26, *p* < 0.01), train period (χ² = 4.81, *p* < 0.01).

Cluster 5, named “COVID-19 Outbreak and health providers motivation and commitment”, accounts for 18.7% of the sample, including literature already focused on the pandemic COVID-19, emphasizing notions of urgency and challenging demands and conditions. Although nurses are the specific health professionals more associated with this cluster, more generic mentions of professionals, providers, and organizational contexts are made. Moreover, the focus on health providers considers their motivation, commitment, and willingness, especially those on the frontline. The main geographical contexts are China, Japan, and Australia. The specific words more associated with this cluster are COVID (χ² = 25.75, *p* < 0.01), nurse (χ² = 41.38, *p* < 0.01), China (χ² = 22.34, *p* < 0.01), professional (χ² = 21.59 *p* < 0.01), work (χ² = 20.03, *p* < 0.01), challenge (χ² = 18.74, *p* < 0.01), influence (χ² = 17.51, *p* < 0.01), perspective (χ² = 27.03, *p* < 0.01), willingness (χ² = 15.34, *p* < 0.01), decision (χ² = 13.94, *p* < 0.01), quality (χ² = 13.14, *p* < 0.01), outbreak (χ² = 10.22, *p* < 0.01), frontline (χ² = 9.22, *p* < 0.01), evidence (χ² = 8.43, *p* < 0.01), natural (χ² = 8.43, *p* < 0.01), provider (χ² = 7.98, *p* < 0.01), disease (χ² = 7.16, *p* < 0.01), care (χ² = 7.05, *p* < 0.01), crisis (χ² = 6.89, *p* < 0.01), patient (χ² = 6.89, *p* < 0.01), condition (χ² = 5.68, *p* < 0.01), demand (χ² = 5.68, *p* < 0.01), motivation (χ² = 5.68, *p* < 0.01), commitment (χ² = 5.68, *p* < 0.01), Australian (χ² = 5.68, *p* < 0.01), problem (χ² = 4.58, *p* < 0.01), policy (χ² = 4.58, *p* < 0.01), organizational (χ² = 4.58, *p* < 0.01), Japan (χ² = 4.58, *p* <0.0), pressure (χ² = 4.58, *p* <0.0), affect (χ² = 4.16, *p* <0.0) and healthcare (χ² = 3.85, *p* <0.0). In order to better grasp the contextual meaning of the concepts pertaining to this cluster, we have consulted the typical text segments in which some of the most frequent words appear (using as ranking score the sum of χ² = of marked forms in segment).

The aggregated thematic patterns indicate what are the main trends of research on public health emergencies and, considering their variance percentage, what are the more or less researched issues. As mentioned, clusters 4 and 5 are those with less salience, referring precisely to the coping and motivation of health providers, nurses, and doctors, during the pandemic COVID-19. On the bright side, the psychological impacts of emergency work, including during the pandemic, have received more attention. One may argue that both topics are intertwined, especially in regards to the role of psychological impact and coping strategies in the motivation of workers [30]. These results provide cues on which topics may need further investment and which articulations can be made to advance a more concerted knowledge on the under-researched theme of willingness to report to public health emergencies.

As shown in Table 7 below, the analysis of the most frequent words/themes by field indicates some interesting patterns: though work is a notion transversal to all fields, the worker is particularly emphasized by psychology journals. Pandemic is also a focus of the three areas, but articles on COVID-19 were only published in medicine and psychiatry journals. The same is true for willingness, risk, disease, and outbreak, very much focused on immediate interventions. Disasters, emergency, influenza, preparedness, and workforce are topics exclusive to medical journals. When the focus is personnel or public (impact at large), psychology is the main area.

The analysis of the most frequent expressions (Figure 6 below) and themes per year (Table 8) reinforces the responsiveness of this literature to the emergency or threat event at hand. It also shows what type of sample (e.g., doctors, nurses) and angle (health staff personnel, administrative, or management) is more salient per year.

## 4. Final Remarks and Conclusions

The literature on the topic of health professionals’ willingness to work under emergency contexts gained traction in the 1980s and, so far, encompasses five main research vectors, as shown by the hierarchical cluster descending analysis: “High Profile Health hazards”, “Psychological impacts of emergency work”, “Outbreaks impact on public healthcare system and workforce”, and two specifically related to COVID-19: “Outbreak and health providers’ motivation and commitment” and “Medical doctors coping”. It is clear that the pandemic COVID-19 was the more addressed issue and almost equally by the three fields: medicine, psychiatry, and psychology. Research within the context of influenza and natural disasters were also common topics, emphasizing their impacts on public healthcare systems and workforces. As shown by the bibliometric data, the USA, China, U.K., and Germany are the more common countries of first authors. The majority of the studies are conducted by researchers from universities, but there is also hospital-based research. Despite the diversity of health-related professions addressed in the studies, medical doctors and nurses are the main health staff actors and, as such, the most commonly researched.

As for the variables that affect willingness or lack thereof to work in emergency contexts, the content analysis showed that there are several embodying ethical dilemmas posed in cases of infectious disease threats: to protect themselves and their family or to respect their professional oath and duty/abide by civic responsibility of saving lives considering their expertise. Socio-demographic factors (age, sex) are alongside the specific hierarchical role, the schedule required, and the quality of preparation training. Belief in duty and belief in protecting their family are at the different symbolic and values-based poles of this dilemma. The individual aptitude for working and thriving in an emergency context depends on the individual risk perception and underlying factors, the family support in engaging in that activity, and the required perceived skills to fight the emergence. Naturally, different provisions are required depending on health emergencies, and the response outlining must consider the aptitudes and willingness of health professionals as well as the available resources in the institution [19]. Specifically, this may involve managing the limitations and fears of each health professional through training and educational experiences paired with a consensual and co-constructed emergency plan where all are aware of what may be required and expectable. It matters to highlight that, despite the perceived barriers and high-risk perception of these events, there are several factors pushing forth their willingness to work in an emergency to public threats. The ways in which the same factors may act as enablers or barriers to willing and committed health professionals suggest the importance of a tailored assessment in each healthcare facility. To know what health professionals value more and what requires additional training, supervision, or other comprehensive measures is fundamental to enhance their preparedness. Concretely, our systematic analysis shows that the factor that matter the most for health professionals to be or not willing are specific socio-demographics (being younger, male and with no family), which means that additional efforts should be made in the training, facilitation and commitment enabling of the remaining age and sex groups; the specific role, responsibilities and duties that must be well defined and clear for all health professionals within a given system so the intervention runs smoothly and with no unnecessary overlapping; schedule, especially if it is too disruptive or intense; belief in duty and ethics of care by the health professional (that should be accessed previously accompanied by raising awareness of its importance); preparation/training, fundamental to put in practice so professionals may gain sense of competence and control of protocols, emergency measures, specific procedures; the type of catastrophe; working conditions that include a wide array of specificities that should be well negotiated between health professionals and healthcare facilities management in articulation with tailored health public policies design.

The mindset, as profusely emphasized during times of crises, should be to capacitate health professionals with the best resources possible, including specific training, psychological accompaniment during and after the exposure to the acute or long-standing event, awareness-raising regarding their role and duties, a clear, comprehensive attitude of support to mitigate the feelings of concern or fear regarding one’s or family health. To know the factors and apply contingency measures is halfway through more capable and willing health professionals to deal with threatening situations with the least impact possible on their health in its aftermath.

## Figures and Tables

**Table 1:** Patterns of authors, institution, research area, citations, and H-index (Scopus).

Author	N	Institution	Research Area	Citations	H-Index
Adams L.M	3	Texas Christian University	Health Sciences	2	6
Alwidyan MT.	2	Jordan University	Science and Technology	0	3
Charney R.	2	St. Louis University School of Medicine	Medicine	12	6
Dieltiens G.	2	Prins Leopold Instituut voor Tropische Geneeskunde	Medicine	5	9
Flood RG.	2	Saint Louis University	Emergency Medicine	14	17
Links J.M.	2	University of Delaware	Engineering	85	47
Magda L.A.	2	Fairleigh Dickinson University	Medicine	23	8
NcNeill C.	2	East Carolina University	Medicine	3	5
Mortelmans L.J.M.	2	University of Leuven	Emergency Medicine	12	12
Rutkow L.	2	Johns Hopkins Bloomberg School of Public Health	Health Policy and Management	3	17
Rebmanm T.	2	Institute for Biosecurity	Epidemiology and Biostatistics	3	19
Sabbe M.B.	2	University Hospital Gasthuisberg	Emergency Medicine	12	24
Smith E.	2	Hotchkiss Brain Institute	Medicine		97
Taylor HA.	2	Johns Hopkins Bloomberg School of Public Health	Health Policy and Management	3	35
Thompson C.B.	2	Johns Hopkins Bloomberg School of Public Health	Biostatistics	70	30

**Table 3:** Scientific journal ranking.

Journal	Number of Articles	Field of Studies	Quartile (2020)
Disaster Medicine and Public Health Preparedness	7	Public health, Environmental and Occupational health	Q2
American Journal of Disaster Medicine	6	Medicine	Q4
International Journal of Environmental Research and Public Health	5	Environmental Science, Medicine	Q1
Plos One Journal	4	Multidisciplinary	Q1
Journal of Emergency Management	3	Engineering, Medicine, and Social Sciences	Q2
Journal of Nursing Management	3	Leadership and Management	Q1
Prehospital Emergency Care	2	Emergency Medicine, Emergency Nursing	Q1
Risk Management and Health Care Policy	2	Medicine	Q2
Journal Clinical Sleep Medicine	2	Medicine and Neuroscience	Q1
Journal of Public Health Management & Practice	2	Health Policy and Public Health, Environmental and Occupational Health	Q2
International Emergency Nursing	2	Emergency Nursing	Q1
Journal of Occupational and Environmental Medicine	2	Medicine	Q2
Prehospital and Disaster Medicine	2	Medicine, Nursing	Q1
BMC Public Health	2	Medicine	Q1

**Table 4:** Research degree of internationalization and interdisciplinarity.

Number of Countries	Number of Studies	Number of Institutions/Departments	Number of Studies
1	118	1	60
2–3	15	2–3	47
4–5	2	4–5	18
>5	2	>5	12

**Table 5:** Type of professionals and health care providers.

Type of Professionals and Health Care Providers	References
Doctors	[22,23,25,26,28,31,32,33,34,35,36,37,38,39,40,41,42,43,44,45,46,47,48,49,50,51,52]
Healthcare assistants	[23,26,43,44,45,50,51,53,54]
Nurses	[22,25,26,35,43,50,51,52,55,56,57,58,59,60,61,62,63,64,65,66,67,68,69,70,71,72,73,74,75,76,77,78,79,80,81,82,83,84,85,86,87,88,89,90,91,92,93,94]
Students	[40,47,48,64,95,96,97,98,99,100,101,102]
Administrative/healthcare managers	[12,22,33,39,55,103]
Pharmacists	[26,37,42,84,96]
Lab personal	[26,37]
Paramedics	[35,47,104]
Workers/staff- generic	[105,106,107,108,109,110,111,112,113,114,115,116,117,118,119,120,121,122,123,124,125,126,127,128,129,130,131,132,133,134,135,136,137,138,139,140,141,142,143,144,145]
Radiological technologists	[26]
Physical therapists	[26]

**Table 7:** Distribution of words and areas.

Content	Medicine	Psychiatry	Psychology
Work	23.46%	21.24%	14.08%
Pandemic	17.47%	24.51%	21.13%
Willingness	16.9%	13.07%	0
Disaster	13.43%	0	0
Work	23.46%	21.24%	14.08%
Health	12.86%	31.05%	28.17%
Worker	10.76%	8.17%	35.21%
COVID	10.68%	27.78%	0
Nurse	10.27%	1.63%	7.04%
Healthcare	9.62%	1.63%	14.08%
Emergency	7.68%	0	0
Influenza	6.47	0	0
Medical	5.99%	1.63%	7.04%
Preparedness	4.04%	0	0
Personnel	4.04%	0	7.04%
Risk	3.8%	1.63%	0
Duty	3.72%	1.63%	0
Public	3.64%	8.17%	14.08%
Disease	3.32%	1.63%	0
Outbreak	2.83%	1.63%	0
Workforce	2.1%	0	0

**Table 8:** Thematic evolution per year: 5 most frequent themes per year of publication.

	Order of Appearance				
Year	1st	2nd	3rd	4th	5th
1989	Personnel	Duty	Disaster	Hospital	Volunteer
2004	Nurse	Preparedness	Readiness	Critical	Work
2005	Care	Health	Disaster	Willingness	Outbreak
2006	Professional	Hospital	Employee	Family	Influenza
2007	Provider	Work	Emergency	Incident	Mass
2008	Hospital	Personnel	Influenza	Pandemic	Respond
2009	Pandemic	Influenza	Worker	Disaster	Healthcare
2010	Pandemic	Health	Influenza	Willingness	Emergency
2011	Pandemic	Care	Paramedic	Influenza	Willingness
2012	Disaster	Healthcare	Willingness	Personnel	Public
2013	Nurse	Disaster	Pandemic	Willingness	Influenza
2014	Disaster	Health	Preparedness	Emergency	Response
2015	Worker	Influenza	Care	Emergency	Preparedness
2016	Staff	Hospital	Medical	Nurse	Outbreak
2017	Emergency	Infectious	Response	Willingness	Preparedness
2018	Firefighter	Personnel	Family	Health	Duty
2019	Willingness	Work	Nurse	Condition	Threat
2020	Pandemic	Willingness	Disaster	COVID	Healthcare
2021	COVID	Work	Pandemic	Willingness	Worker

## Data Availability

Not applicable.

## Supplementary Materials

The following are available online at https://www.mdpi.com/article/10.3390/healthcare10081500/s1, Table S1: The main global information collected from the publications. [8,10,11,12,19,20,21,22,23,24,25,26,27,28,31,32,33,34,35,36,37,38,39,40,41,42,43,44,45,46,47,48,49,50,51,52,53,54,55,56,57,58,59,60,61,62,63,64,65,66,67,68,69,70,71,72,73,74,75,76,77,78,79,80,81,82,83,84,85,86,87,88,89,90,91,92,93,94,95,96,97,98,99,100,101,102,103,104,105,106,107,108,109,110,111,112,113,114,115,116,118,119,120,121,122,123,124,125,126,127,128,129,130,131,132,133,134,135,136,137,138,139,140,141,142,143,144,145,146,147,148,149,150,151,152,153,154,155,157,158,159] are cited in the supplementary.
