# Supplementary material for: Willingness to Work during Public Health Emergencies: A Systematic Literature Review
Source: Healthcare (Basel). 2022 Aug 9;10(8):1500. doi: 10.3390/healthcare10081500 (PMC9408569; doi:10.3390/healthcare10081500)
Supplement: Supplementary file 1 [file healthcare-10-01500-s001.zip › healthcare-1819055-supplementary.pdf]

Table S1. The main global information collected from the publications.

Author	Year	Title	Journal	Aim
Adams, L.M. [93]	2009	Surge Readiness	*Nurse Leader*	To understand how off-duty staff members will report to work if called in, in the event of a disaster.
Adams, L.M.; Berry, D. [67]	2012	Who will show up? Estimating ability and willingness of essential hospital personnel to report to work in response to a disaster	*Online journal of issues in nursing*	To discuss the need for essential personnel following a disaster, review the literature related to adequate disaster staffing, and share the study examining both the ability and willingness of healthcare personnel to report to work during a disaster.
Adams, L.M.; Melius, J. [57]	2020	Prepared to Respond? Exploring Personal Disaster Preparedness and Nursing Staff Response to Disasters	*Disaster medicine and public health preparedness*	To explore personal disaster preparedness of nursing staff and their ability and willingness to respond following a disaster.
Al-Dorzi, H.M. et al. [28]	2016	The critical care response to a hospital outbreak of Middle East respiratory syndrome coronavirus (MERS-CoV) infection: an observational study	*Ann Intensive Care*	To learn from the experience regarding the critical care response to the outbreak.
Al-Hunaishi, W.; Hoe, V. C.; Chinna, K. [94]	2019	Factors associated with healthcare workers willingness to participate in disasters: a cross-sectional study in Sana'a, Yemen	*BJM Open*	To assess healthcare workers willingness to participate in biological and natural disasters, and to identify its associated factors.

Almaghrabi, R. H. et al. [31]	2020	Healthcare workers experience in dealing with Coronavirus (COVID-19) pandemic.	*Saudi medical journal*	To analyze healthcare workers experiences in dealing with the Coronavirus (COVID-19) pandemic.
Al-Ma'seb, H.; Alaslawi, H. A. [119]	2021	Social Workers' Knowledge and Ability in Crisis Intervention in Kuwait- A Case of COVID-19	*Social work in public health*	To assess whether there are significant differences among variables, such as social workers' familiarity with the crisis intervention model, receiving up-to-date information about the latest developments in COVID-19.
Al Muharraq E. H. [83]	2021	The Psychological Impact of Coronavirus Disease 2019 on Nurses in Saudi Arabia and Their Coping Strategies.	*SAGE open nursing*	To explore the psychological impact of coronavirus disease 2019 on frontline nurses working in Jazan, Saudi Arabia, and their coping strategies.
Alwidyan, M. et al. [35]	2021	Are healthcare professionals in Jordan willing to work and provide care for COVID-19 patients?	*Australasian Journal of Paramedicine*	To assess the willingness of healthcare providers in Jordan to report for duty and provide care to COVID-19 patients.
Alwidyan, M. T.; Oteir, A. O.; Trainor, J. [36]	2020	Working During Pandemic Disasters: Views and Predictors of EMS Providers.	*Disaster medicine and public health preparedness*	To assess the perception and attitude of emergency medical services (EMS) providers toward working during disease outbreaks, and the factors that may influence their decisions to ultimately work or not.
Alwidyan, M. T.; Trainor, J. E.; Bissell, R. A. [34]	2020	Responding to natural disasters vs. disease outbreaks: Do emergency medical service providers have different views?	*International journal of disaster risk reduction*	To assess a sample of U.S EMS providers' views about working during natural disasters and disease outbreaks and explore whether they are coming to work during such conditions.
Al-Shaqsi, S. et al. [68]	2015	Self-reported preparedness of New Zealand acute care providers to mass emergencies before the	*Emergency medicine Australasia: EMA*	To assess the self-reported training and experience of New Zealand acute care providers to respond to mass emergencies and the factors associated with strong preparedness.

		Canterbury Earthquakes: a national survey.		
Al-Wathinani, A. M. et al. [69]	2021	A Cross-Sectional Study on the Flood Emergency Preparedness among Healthcare Providers in Saudi Arabia.	*International journal of environmental research and public health*	To measure healthcare workers' knowledge, attitudes, perceptions, and willingness to respond to a flood scenario in Saudi Arabia.
Aoyagi, Y. et al. [27]	2015	Healthcare workers' willingness to work during an influenza pandemic: a systematic review and meta-analysis.	*Influenza and other respiratory viruses*	To estimate the proportion of healthcare workers (HCWs) willing to work during an influenza pandemic and identify associated risk factors.
AlSaif, H. I. et al. [95]	2020	Willingness and Self-Perceived Competence of Final-Year Medical Students to Work as Part of the Healthcare Workforce During the COVID-19 Pandemic.	*International journal of general medicine*	To explore the willingness of final-year medical students to meet this demand, examine their perceived competence, and determine how their overall perceived competence correlates with their willingness.
Arbon, P. et al. [87]	2013	Exploring staff willingness to attend work during a disaster: a study of nurses employed in four Australian emergency departments.	*Australasian emergency nursing journal: AENJ*	To explore nurses' willingness to attend work during a disaster and the factors that influenced this decision.
Balicer, R.D. et al. [25]	2010	Characterizing hospital workers' willingness to report to duty in an influenza pandemic through threat- and efficacy-based assessment.	*BMC Public Health*	To report to work during an influenza pandemic is a critical yet under-studied phenomenon.
Barnett D.J. et al. [24]	2009	Assessment of Local Public Health Workers' Willingness to Respond to Pandemic Influenza through	*PLOS ONE*	To use the EPPM as a lens for examining the influences of perceived threat and efficacy on local public health workers' response willingness to pandemic influenza.

		Application of the Extended Parallel Process Model		
Barqawi H.J. et al. [48]	2021	Readiness of physicians and medical students to cope with the COVID-19 pandemic in the UAE.	*PLOS ONE*	To assess the knowledge, preparedness and attitudes of medical students, physicians and faculty members in the United Arab Emirates (UAE) on COVID-19 and their perspective on the roles of educational and healthcare institution towards improving pandemic preparedness and enabling optimal care.
Basta, N. E.; Edwards, S. E.; Schulte, J. [91]	2009	Assessing public health department employees' willingness to report to work during an influenza pandemic.	*Journal of public health management and practice*	To determine how informed health department employees are about pandemic response and how willing they are to report to work during a pandemic.
Bell, M. A. et al. [77]	2014	A national survey of emergency nurses and avian influenza threat.	*Journal of emergency nursing*	To determine the perceived likelihood of emergency nurses reporting to work during an avian influenza outbreak, to consider options if nurses decided not to report work, and to explore Protection Motivation Theory constructs as predictors of reporting to work.
Ben Natan, M. et al. [70]	2014	Nurse willingness to report for work in the event of an earthquake in Israel.	*Journal of nursing management*	To examine variables affecting nurse willingness to report for work in the event of an earthquake in Israel and whether this can be predicted through the Theory of Self-Efficacy.
Braune, K. et al. [124]	2021	Interdisciplinary Online Hackathons as an Approach to Combat the COVID-19 Pandemic: Case Study.	*Journal of medical Internet research*	To describe the steps and methods employed in the conduction of a remote online health hackathon centered on challenges posed by the COVID-19 pandemic.
Brice, J. H. et al. [71]	2017	Survey of Hospital Employees' Personal Preparedness and Willingness to Work Following a Disaster.	*Southern medical journal*	To understand the personal disaster preparedness for hospital staff.

Charney, R. L.; Rebmann, T.; Flood, R. G. [8]	2015	Hospital Employee Willingness to Work during Earthquakes Versus Pandemics.	*The Journal of emergency medicine*	To understand what is to work during a pandemic and that the willingness of nonlicensed staff to work has had limited assessment.
Charney, R.; Rebmann, T.; Flood, R. G. [153]	2014	Working after a tornado: a survey of hospital personnel in Joplin, Missouri.	*Biosecurity and bioterrorism: biodefense strategy, practice, and science*	To assess the resilience of healthcare workers in hospitals as measured by number reporting to work, willingness to work, personal disaster preparedness, and childcare responsibilities following the disaster.
Chow, C. et al. [45]	2021	Enhancing frontline workforce volunteerism through exploration of motivations and impact during the COVID-19 pandemic.	*International Journal of Disaster Risk Reduction*	To explore the motivational factors underlying healthcare worker volunteerism in COVID-19 pandemic response operations in the community, and to describe the impact of such operations on volunteers, in order to highlight important volunteer recruitment strategies and ensure volunteer sustainability.
Chaudhary, F. A. et al. [122]	2021	The psychological impact of the covid-19 pandemic on oral health care workers and its impact on their willingness to work during this pandemic.	*Archives of Psychiatry Research*	To investigate the relationship between the psychological status of oral health care workers (OHCWs) and the willingness to treat a patient during the COVID-19 pandemic.
Coberly, E. et al. [123]	2021	Implementation of measures to improve blood collection center employee safety and reduce callouts during the 2019 novel coronavirus pandemic.	*Transfusion*	To determine the impact of safety measures including social distancing, masks, enhanced disinfection protocols, and temperature screening in blood collection centers employee.
Cone, D. C.; Cummings, B. A. [61]	2020	Hospital disaster staffing: If you call, will they come?	*American journal of disaster medicine*	To assess hospital employees' attitudes and needs regarding work commitments during disasters.
Connor S. B. [62]	2014	Factors associated with the intention of health care personnel to respond to a disaster.	*Prehospital and disaster medicine*	To understand how to respond when a disaster threatens the health of a community.
Conroy, D. A. et al. [125]	2021	The effects of COVID-19 stay-at-home order on sleep, health, and	*Journal of clinical sleep medicine: JCSM*	To explore changes in sleep, health, work, and mood in health care workers during the stay-at-home orders.

		working patterns: a survey study of US health care workers.		
Cowden, J. et al. [144]	2010	Pre-pandemic planning survey of healthcare workers at a tertiary care children's hospital: ethical and workforce issues.	*Influenza and other respiratory viruses*	To determine the relationship between healthcare worker (HCW) reporting willingness to work during a pandemic and perception of job importance, belief that one will be asked to work, and sense of professionalism.
Crowley J. [103]	2020	Disaster debris management best practice case studies.	*Journal of emergency management*	To identify debris management best practices for planning and responding to debris-generating disaster events.
Csigó K; Ritzl A. [126]	2021	First reactions and attitudes of psychiatric workers in Budapest psychiatric care units regarding the COVID-19 pandemic.	*Prim Care Companion CNS Disord*	To focus on the first feelings, attitudes, and planned reactions of psychiatric workers in Hungary to the news of the appearance of coronavirus disease 2019 (COVID-19) and to examine any differences between psychiatrists/psychologists and nurses/other professionals regarding these attitudes.
Dalton, C. B.; Durrheim, D. N.; Conroy, M. A. [150]	2008	Likely impact of school and childcare closures on public health workforce during an influenza pandemic: a survey.	*Communicable diseases intelligence quarterly report*	To work due to illness, transport disruptions or care responsibilities, including care of children if school closures occur.
Damery, S. et al. [92]	2009	Will the NHS continue to function in an influenza pandemic? a survey of healthcare workers in the West Midlands, UK.	*BMC Public Health*	To attend work during a pandemic are subject to scientific and predictive uncertainty, yet an informed evidence base is needed if contingency plans addressing the issues of HCW absenteeism are to be prepared.
Daugherty, E. et al. [52]	2009	Survey Study of the Knowledge, Attitudes, and Expected Behaviors of Critical Care Clinicians Regarding an Influenza Pandemic.	*Infection Control & Hospital Epidemiology*	To characterize the knowledge, attitudes, and expected behaviors of ICU HCWs concerning the risk of and response to an influenza pandemic.
Deng, J. et al. [101]	2021	Effects of COVID-19 on career and specialty choices among Chinese medical students.	*Medical education online*	To investigate the effects of COVID-19 pandemic on medical career and specialty choices among medical students.
DeSimone, C. L. [157]	2009	Response of Public Health Workers to Various Emergencies.	*AAOHN Journal*	To measure public health workers' opinions on disaster preparedness, assess workers' likelihood of reporting to

				various types of disasters, and evaluate conditions that will encourage workers to report to work.
Devnani M. [145]	2012	Factors associated with the willingness of health care personnel to work during an influenza public health emergency: an integrative review.	*Prehospital and disaster medicine*	To determine the state of the evidence concerning the willingness of HCP to work during an influenza public health emergency, to identify the gaps for future investigation, and to facilitate evidence-based influenza public health emergency planning.
Dhont, J. et al. [127]	2020	Conducting research in Radiation Oncology remotely during the COVID-19 pandemic: Coping with isolation.	*Clinical and translational radiation oncology*	To carried out a survey amongst researchers in the field of radiation oncology to gain insights on the impact of social isolation and working from home and to guide future work.
Dickinson, J. A. et al. [41]	2013	Alberta family physicians' willingness to work during an influenza pandemic: a cross-sectional study.	*Asia Pacific family medicine*	To investigate Alberta family physician's willingness to work during an influenza pandemic.
Diel, R.; Hittel, N.; Nienhaus, A. [108]	2021	Point-of-Care COVID-19 Antigen Testing in Exposed German Healthcare Workers-A Cost Model.	*International journal of environmental research and public health*	To assess the costs of sending HCW into quarantine (Scenario 1) from the hospital's and the taxpayer's perspective versus the costs arising from implementing point-of-care COVID-19 antigen testing (POCT) for those staff members who, despite learning that they have been exposed to hospital patients later found to be infected with COVID-19, continue to report to work (Scenario 2).
Dreher, A. et al. [46]	2021	Attitudes and stressors related to the SARS-CoV-2 pandemic among emergency medical services workers in germany: A cross-sectional study.	*BMC Health Services Research*	To investigate attitudes and stressors related to the SARS-CoV-2 outbreak among emergency medical services (EMS) workers in Germany.
Dutta, U. et al. [128]	2021	Multidimensional dynamic healthcare personnel (HCP)-centric model from a low-income and middle-income country to support	*BMJ open*	To implement a novel multidimensional HCP-centric evidence-based, dynamic policy with the objectives to reduce risk of HCP infection, ensure welfare and safety of

		and protect COVID-19 warriors: a large prospective cohort study.		the HCP and to improve willingness to accept and return to duty.
Ehrenstein, B.P.; Hanses, F.; Salzberger, B. [22]	2006	Influenza pandemic and professional duty: family or patients first? A survey of hospital employees.	*BMC Public Health*	To survey employees at a university hospital regarding ethical issues arising during the management of an influenza pandemic.
Foley, D. A. et al. [33]	2020	COVID-19 and paediatric health services: A survey of paediatric physicians in Australia and New Zealand.	*Journal of paediatrics and child health*	To assess attitudes, readiness and confidence in the early stages of the COVID-19 pandemic through an online survey of pediatric physicians and sub-specialists across Australia and New Zealand.
Fung, O. W.; Loke, A. Y. [63]	2013	Nurses' willingness and readiness to report for duty in a disaster.	*Journal of emergency management*	To seeks for a better understanding of the factors affecting nurses' willingness and readiness to report for duty in a disaster.
Ganz, F. D. et al. [72]	2019	A Conflict of Values: Nurses' Willingness to Work Under Threatening Conditions.	*Journal of nursing scholarship: an official publication of Sigma Theta Tau International Honor Society of Nursing*	To determine the willingness of Israeli registered nurses to work under threatening conditions, their perceived level of threat, and perceptions of peer willingness to report to work.
Garcia, A. S. et al. [81]	2021	Parenting stress, self-efficacy and COVID-19 health risks as predictors of general stress among nurses.	*International Journal of Nursing Practice*	To examine the influence of parenting stress, self-efficacy and COVID-19 health risks on general stress among nurses in the Midwest, United States, during the pandemic.
Garrett, A.; Park, Y.; Redlener, I. [137]	2009	Mitigating Absenteeism in Hospital Workers During a Pandemic.	*Disaster Medicine and Public Health Preparedness*	To mitigate absenteeism in hospital workers during a pandemic.
Gershon, MHS et al. [109]	2010	Pandemic-related ability and willingness in home healthcare workers.	*American Journal of Disaster Medicine*	To assess pandemic-related attitudes and behavioral intentions of home healthcare workers (HHCWs).
Gershon, R. R. et al. [110]	2011	Mass fatality preparedness in the death care sector.	*Journal of occupational and*	To characterize mass fatality preparedness of the death care sector (ie, funeral industry organizations) and to determine

			environmental medicine	the workforce's ability and willingness to report to duty during a hypothetical high fatality pandemic event.
Goh, E.; Baum, T. [129]	2021	Job perceptions of Generation Z hotel employees towards working in Covid-19 quarantine hotels: the role of meaningful work.	*International Journal of Contemporary Hospitality Management*	To investigate the motivations that young hotel employees hold towards working in a potentially dangerous hotel workplace.
Goodhue, C. J. et al. [143]	2012	Willingness to respond in a disaster: a pediatric nurse practitioner national survey.	*Journal of pediatric health care: official publication of National Association of Pediatric Nurse Associates & Practitioners*	To examine factors associated with pediatric nurse practitioners (PNPs) reporting to work in the event of a disaster.
Goni-Fuste, B. et al. [138]	2021	Experiences and needs of nursing students during pandemic outbreaks: A systematic overview of the literature.	*Journal of professional nursing: official journal of the American Association of Colleges of Nursing*	To conduct a comprehensive systematic overview of the literature concerning nursing students in the context of emerging infectious disease epidemics or pandemics caused by zoonotic viruses.
Han, S. et al. [47]	2021	Associations between the working experiences at frontline of COVID-19 pandemic and mental health of korean public health doctors.	*BMC Psychiatry*	To find predictors of mental health for public health doctors from working experiences at frontline of COVID-19 pandemic.
Hernández-Martínez, A. et al. [98]	2021	Nursing students' experience and training in healthcare aid during the COVID-19 pandemic in Spain.	*Journal of clinical nursing*	To face the situation, and what must be improved in their training to be ready for these situations is unknown.
Hill, M.; Smith, E.; Mills, B. [105]	2021	Willingness to Work amongst Australian Frontline Healthcare Workers during Australia's First Wave of Covid-19 Community Transmission: Results of an Online Survey.	*Disaster medicine and public health preparedness*	To work during Australia's first wave of the COVID-19 pandemic among frontline HCWs.

Hope, K. [139]	2010	Willingness of frontline health care workers to work during a public health emergency.	*The Australian Journal of Emergency Management*	To determine the perceived willingness to report to work during three public health emergency scenarios (weather event, influenza pandemic and bioterrorism event).
Imai, H. et al. [113]	2010	Factors associated with motivation and hesitation to work among health professionals during a public crisis: a cross sectional study of hospital workers in Japan during the pandemic (H1N1) 2009.	*BMC Public Health*	To maintain hospital function under critical situations such as a pandemic, it is important to understand the factors that increase and decrease the willingness to work.
Inal, E.; Kaya, E. [64]	2021	Health care students' willingness to work during disasters in Turkey.	*International emergency nursing*	To explore the willingness of health care students in nursing, emergency and disaster management (EDM) departments to work during disasters and to examine sociodemographic and disaster-related factors that affect their willingness to work during disasters.
Irvin, C. et al. [53]	2008	Survey of Hospital Healthcare Personnel Response during a Potential Avian Influenza Pandemic: Will They Come to Work?	*Prehospital and Disaster Medicine*	To survey hospital personnel regarding their attendance at work in the hypothetical event of avian influenza pandemic, and what factors might influence this decision.
Iserson K. V. [151]	2020	Healthcare Ethics During a Pandemic.	*The western journal of emergency medicine*	To discuss how healthcare professionals weigh risk factors related to their response and the actions the healthcare community can take, including proper communication and mitigating responder concerns, to maximize and maintain our caregiver workforce
Ives, J. et al. [21]	2009	Healthcare workers' attitudes to working during pandemic influenza: a qualitative study.	*BMC Public Health*	To cope during an influenza pandemic will depend, to a large extent, on the number of HCWs who are able and willing to work through the crisis.
Iyama, K. et al. [78]	2020	Factors associated with the intention to participate in activities	*Journal of radiation research*	To identify the factors affecting the intention to participate in nuclear disaster activities.

		during a nuclear disaster situation among firefighters.		
Jang, Y. et al. [43]	2021	Burnout and peritraumatic distress of healthcare workers in the COVID-19 pandemic.	*BMC Public Health*	To evaluate the current status of emotional exhaustion and peritraumatic distress of healthcare workers (HCWs) in the COVID-19 pandemic, and identify factors associated with their mental health status.
Jang, Y. et al. [44]	2021	Factors Associated with the Work Intention of Hospital Workers' in South Korea During the Early Stages of the COVID-19 Outbreak.	*Disaster medicine and public health preparedness*	To explore factors associated with the work intention of hospital workers in the early stages of the coronavirus disease (COVID-19) outbreak in South Korea.
Kagan, I.; Itzhaki, M.; Melnikov, S. [59]	2017	Patriotism, organizational commitment and nurses' intention to report for work in emergencies.	*International nursing review*	To examine the influence of nurses' patriotism and organizational commitment on their intention to report for work in a national emergency, in Israel.
Karki, P. et al. [99]	2021	Willingness of nepalese medical and nursing students to volunteer during COVID-19 pandemic: A single-centered cross-sectional study.	*Annals of Medicine and Surgery*	To know the willingness of medical and nursing students to volunteer during the coronavirus disease-19 (COVID-19) pandemic.
Katz, S. K. et al. [146]	2014	Public health aspects of nuclear and radiological incidents.	*American journal of disaster medicine*	To identify the public health aspects of nuclear and radiological incidents and prevent or mitigate such events.
Kaya, E.; Altintas, H. [65]	2018	Willingness of Firefighting Program Students to Work in Disasters-Turkey.	*Prehospital and disaster medicine*	To understand the willing personnel to work in disasters.
Ke, Q. et al. [60]	2021	Frontline nurses' willingness to work during the COVID-19 pandemic: A mixed-methods study.	*Journal of advanced nursing*	To examine frontline nurses' willingness to work, identify its predictors and explore its corresponding reasons.
Khalid, M. et al. [40]	2021	Risk Perception and Willingness to Work Among Doctors and Medical Students of Karachi, Pakistan	*Risk management and healthcare policy*	To determine the factors that influence the willingness of HCWs to work during a pandemic, in order to effectively

		During the COVID-19 Pandemic: A Web-Based Cross-Sectional Survey.		plan, prepare, and ensure the continued delivery of essential health services during a pandemic.
Koh, T.J.W. et al. [100]	2021	Attitudes towards COVID-19 precautionary measures and willingness to work during an outbreak among medical students in Singapore: a mixed-methods study.	*BMC Med Educ*	To assess medical students' education needs in pandemic preparedness.
Liu, Z. et al. [147]	2020	I Don't Want to Go Back: Examining the Return to Physical Workspaces During COVID-19.	*Journal of occupational and environmental medicine*	To inform employers' and policy makers' decision making around the return to work during COVID-19.
Loke, A. Y.; Fung, W. M.; Liu, X. [66]	2013	Mainland China nurses' willingness to report to work in a disaster.	*Journal of emergency management*	To understand the factors affecting Chinese nurses' willingness to report to work in a disaster.
Luo, Y. et al. [74]	2021	Willingness to participate in front-line work during the COVID-19 pandemic: A cross-sectional study of nurses from a province in South-West China.	*Journal of nursing management*	To explore the current status of Chinese nurses' willingness to work during the COVID-19 pandemic and the factors that influence them.
Martinese, F. et al. [115]	2009	How would Australian hospital staff react to an avian influenza admission, or an influenza pandemic?	*Emergency medicine Australasia: EMA*	To estimate the expected staff absentee rates and work attitudes in an Australian tertiary hospital workforce in two hypothetical scenarios: (i) a single admission of avian influenza; and (ii) multiple admissions of human pandemic influenza.
Ma, X. et al. [51]	2011	Knowledge and attitudes of healthcare workers in Chinese intensive care units regarding 2009 H1N1 influenza pandemic.	*BMC Infect Dis*	To describe the knowledge and attitudes of critical care clinicians during the 2009 H1N1 influenza pandemic.

Maraqa, B.; Nazzal, Z.; Zink, T. [32]	2021	Mixed Method Study to Explore Ethical Dilemmas and Health Care Workers' Willingness to Work Amid COVID-19 Pandemic in Palestine.	*Frontiers in medicine*	To assess Palestinian HCWs' willingness to work and the related factors as well as to explore their ethical dilemmas during the coronavirus disease 2019 (COVID-19) pandemic.
Martin, S. [90]	2010	If they know, will they show? How nurses' knowledge of pandemic flu affects their willingness to work, with implications for home care managers.	*Caring: National Association for Home Care magazine*	To estimate the proportion of healthcare workers (HCWs) willing to work during an influenza pandemic and identify associated risk factors.
Martin, S. D. [88]	2011	Nurses' ability and willingness to work during pandemic flu.	*Journal of nursing management*	To report factors affecting nurses' ability and willingness to work during pandemic flu (PF).
Martin, S. D.; Brown, L. M.; Reid, W. M. [76]	2013	Predictors of nurses' intentions to work during the 2009 influenza A (H1N1) pandemic.	*The American journal of nursing*	To study potential predictors of nurses' intentions to work during the 2009 influenza A (H1N1) pandemic.
Masterson, L. et al. [118]	2009	Willingness to respond: of emergency department personnel and their predicted participation in mass casualty terrorist events.	*The Journal of emergency medicine*	To examine ED personnel willingness to respond to various multiple casualty events.
McNeill, C. et al. [55]	2020	Emergency Preparedness Competencies Among Nurses: Implications for Nurse Administrators.	The Journal of nursing administration	To examine current levels of self-reported professional emergency preparedness competence among nurses.
McNeill, C. et al. [56]	2020	Characterization of nurses' duty to care and willingness to report.	*Nursing ethics*	To determine the willingness to report during disaster events, potentially creating an ethical dilemma and impacting patient care.
Mele, B. S. et al. [42]	2021	Healthcare workers' perception of gender and work roles during the COVID-19 pandemic: A mixed-methods study.	*BMJ Open*	To determine how work and mental health for healthcare workers changed during the COVID-19 pandemic in a universal healthcare system, stratified by gender factors.

Melnikov, S.; Itzhaki, M.; Kagan, I. [86]	2014	Israeli nurses' intention to report for work in an emergency or disaster.	*Journal of Nursing Scholarship*	To investigate the effect of personal characteristics and organizational factors on nurses' intention to report for work in a national emergency.
Mercer, MD et al. [120]	2014	Ready or not: Does household preparedness prevent absenteeism among emergency department staff during a disaster?.	*American Journal of Disaster Medicine*	To determine if the degree of readiness of ED personnel, as measured by household preparedness, is associated with predicted likelihood of reporting for duty.
Minks, C.; Richter, A. [121]	2020	Current State of (Dis)Integration: Public Health and Fusion Centers.	*Journal of Homeland Security and Emergency Management*	To examine the current level of information sharing and integration between these domains by measuring the inclusion of public health in the law enforcement functions of fusion centers
Mitani, S. et al. [26]	2011	Ensuring Adequate Human Medical Resources during an Avian Influenza A/H5N1 Pandemic.	*Prehospital and Disaster Medicine*	To clarify how many medical staff would be willing to work during a pandemic, and requirements to secure adequate human resources.
Mohammadi, F. et al. [130]	2021	Management of COVID-19-related challenges faced by EMS personnel: A qualitative study.	*BMC Emergency Medicine*	To identify some strategies to manage the COVID-19-related challenges faced by the pre-hospital emergency care personnel in the south of Iran.
Montgomery, Z. A. et al. [131]	2021	Are orthopaedic providers willing to work overtime to address COVID-19-related patient backlogs and financial deficits?	*Bone & joint open*	To examine orthopaedic provider preferences regarding the patient care backlog and financial recovery initiatives in response to the COVID-19 pandemic.
Mortelmans, L. J. et al. [10]	2016	Are Tertiary Care Paediatricians Prepared for Disaster Situations?	*Prehospital and disaster medicine*	To evaluate perceived knowledge and capability of tertiary pediatricians to deal with disasters, to identify promoting factors, and to evaluate education need and willingness to work.
Mortelmans, L. J. et al. [102]	2016	Are Belgian military students in medical sciences better educated in disaster medicine than their civilian colleagues?	*J. R. Army Med. Corps*	To evaluate the impact of military training on disaster education in medical science students.

Morris, A. M. et al. [116]	2016	Personal and professional challenges confronted by hospital staff following hurricane sandy: a qualitative assessment of management perspectives.	*BMC emergency medicine*	To assess the personal and professional challenges that affected staff decisions to report to work following a natural disaster and examined the role of management in addressing staff needs and concerns.
Nagel, L. [148]	2020	"The influence of the COVID-19 pandemic on the digital transformation of work".	*International Journal of Sociology and Social Policy*	To investigate whether the COVID-19 pandemic has led to an acceleration of the digital transformation in the workplace.
Natan, M.B. et al. [70]	2014	Nurse willingness to report for work in the event of an earthquake in Israel.	*J. Nurs. Manag.*	To examine variables affecting nurse willingness to report for work in the event of an earthquake in Israel.
Nashwan, AJ. et al. [82]	2021	Nurses' willingness to work with COVID-19 patients: The role of knowledge and attitude	*Nurs Open*	To assess the role of nurses' knowledge and attitude in relation to their willingness to work with patients diagnosed with COVID-19 in Qatar.
Ochi, S. et al. [79]	2016	Hospital Staff Shortage after the 2011 Triple Disaster in Fukushima, Japan-An Earthquake, Tsunamis, and Nuclear Power Plant Accident: A Case of the Soso District.	*PloS One*	To measure this shortage in hospital staff and another is to compare the difference in recovery by hospital types and by categories of hospital staff.
Ogedegbe, C. et al. [80]	2012	Health care workers and disaster preparedness: barriers to and facilitators of willingness to respond.	*International journal of emergency medicine*	To compare perceptions of clinical and non-clinical staff with regard to personal needs, willingness to report (WTR) to work, and level of confidence in the hospital's ability to protect safety and provide personal protective equipment (PPE) in the event of a disaster.
Augustine, J.; Hackman, B.; Kelly, P. [89]	2007	OSHA pandemic guidance reinforces need to plan.	*ED management: the monthly update on emergency department management*	To protect the staff and to prepare for the inevitable increased absenteeism should a pandemic occur.

Park, Y. S. et al. [142]	2015	Do shared barriers when reporting to work during an influenza pandemic influence hospital workers' willingness to work? A multilevel framework.	*Disaster medicine and public health preparedness*	To identify characteristics associated with interventions and barriers that influence health care workers' willingness to report for duty during an influenza.
Patel, R. et al. [96]	2017	Health Care Student Knowledge and Willingness to Work in Infectious Disease Outbreaks.	*Disaster medicine and public health preparedness*	To compare factors influencing Willingness to Work among medical, nursing, and pharmacy students.
Qureshi, K. et al. [20]	2005	Health care workers' ability and willingness to report to duty during catastrophic disasters.	*Journal of urban health: bulletin of the New York Academy of Medicine*	To determine the ability and willingness to report to work during various catastrophic events.
Rafi, M. A. et al. [38]	2021	Willingness to work during initial lockdown due to COVID-19 pandemic: Study based on an online survey among physicians of Bangladesh.	*PloS one*	To determine the willingness and its associated factors to work during the COVID-19 pandemic among the physicians of Bangladesh.
Rebmann, T. et al. [135]	2020	Emergency Medical Services Personnel's Pandemic Influenza Training Received and Willingness to Work during a Future Pandemic.	*Prehospital emergency care*	To identify determinants of emergency medical service (EMS) personnel's willingness to work during an influenza pandemic.
Ritvo, P. et al. [111]	2013	Canadian national surveys on pandemic influenza preparations: pre-pandemic and peri-pandemic findings.	*BMC public health*	To prepared for the future outbreak, partly guided by an ethical framework developed within the Canadian Program of Research on Ethics in a Pandemic (CanPREP).
Ritvo, P. et al. [112]	2020	Canadian survey on pandemic flu preparations.	*BMC Public Health*	To obtain opinions on key ethical issues in pandemic preparedness planning an ethical framework.

Rossow, C. et al. [49]	2013	Healthcare providers: Will they come to work during an influenza pandemic?	*WIT Transactions on the Built Environment*	To examine available evidence on healthcare providers' willingness to report to work during a pandemic influenza and on related measures.
Rutkow, L. et al. [149]	2017	Perceived Facilitators and Barriers to Local Health Department Workers' Participation in Infectious Disease Emergency Responses.	*Journal of public health management and practice: JPHMP*	To understand why local health department workers are willing, or not willing, to report to work during an infectious disease response.
Rutkow, L.; Taylor, H. A.; Powell, T. [39]	2017	Employer Requirements to Work during Emergency Responses: Key Ethics Considerations.	*The Journal of law, medicine & ethics : a journal of the American Society of Law, Medicine & Ethics*	To ensure that employees do report to work.
Seale, H. et al. [23]	2009	"Will they just pack up and leave?" – attitudes and intended behaviour of hospital health care workers during an influenza pandemic.	*BMC Health Serv Res*	To explore HCWs knowledge, attitudes and intended behaviour towards pandemic influenza.
Shapira, S. et al. [75]	2016	Knowledge, perceptions, attitudes and willingness to report to work in an earthquake: A pilot study comparing Canadian versus Israeli hospital nursing staff.	*International emergency nursing*	To respond effectively to an earthquake event and provide lifesaving treatment to an influx of casualties. Understanding the factors that may influence nurses' willingness to report (WTR) in different social contexts and preparedness approaches is crucial for improving preparedness of medical facilities.
Shapira, S. et al. [152]	2019	Healthcare workers' willingness to respond following a disaster: a novel statistical approach toward data analysis.	*BMC medical education*	To respond is an important factor in the health system's response capacity during emergencies.
Shabanowitz, R.B.; Reardon, J.E. [140]	2009	Avian Flu Pandemic – Flight of the Healthcare Worker?	*HEC Forum*	To investigate opinions from healthcare workers themselves on the perceived duty to treat, and how they might respond to a severe avian flu pandemic.

Smith, E. [158]	2014	Willingness to work during a terrorist attack: A case-study of first responders during the 9/11 world trade center terrorist attacks.	*Australasian Journal of Paramedicine*	To response and the importance of the management of terrorist events.
Smith, E.; Burkle, F.; Archer, F. [104]	2011	Fear, Familiarity, and the Perception of Risk: A Quantitative Analysis of Disaster-Specific Concerns of Paramedics.	*Disaster Medicine and Public Health Preparedness*	To explore paramedics' perception of risk and willingness to work, with a specific focus on identifying which type of disasters that paramedics associate with greater levels of fear, familiarity, and risk.
Smith, E. et al. [19]	2009	Paramedics' perceptions of risk and willingness to work during disasters.	*Australian Journal of Emergency Management*	To be both willing and able to respond when disaster strikes, paramedics may be reluctant to work when the situation poses a possible threat to their own safety, their co-workers, or that of their families.
Sproull, M. et al. [159]	2021	The impact of radiation dread on mass casualty medical management during a radiological or nuclear event.	*American Journal of Disaster Medicine*	To create comprehensive emergency planning and preparedness strategies for management of a radiological or nuclear event in the US.
Sultan, M.A. et al. [12]	2021	Impact of virtual disaster collaboration exercises on disaster leadership at hospitals in saudi arabia.	*International Journal of Disaster Risk Science*	measured the impact of virtual three-level collaboration (3LC) exercises on participants' perceived levels of collaboration, learning, and utility (CLU) at hospitals in the southern region of Saudi Arabia.
Sultan, M. et al. [11]	2020	Nurses' Readiness for Emergencies and Public Health Challenges—The Case of Saudi Arabia.	*Sustainability*	To evaluates the perceptions of preparedness and willingness to work during disasters and public health emergencies.
Scully, A. C. et al. [132]	2021	Willingness and ability of oral health care workers to work during the COVID-19 pandemic.	*Journal of the American Dental Association*	To work during the COVID-19 pandemic, barriers to working, and willingness to receive a COVID-19 vaccine.
Stallings, R. A. [141]	1989	Volunteerism Inside Complex Organizations: Off-Duty Hospital Personnel in a Disaster.	*Nonprofit and Voluntary Sector Quarterly*	To work voluntarily at a short-term general hospital treating victims of a major disaster in a metropolitan area.

Stergachis, A. et al. [154]	2011	Health care workers' ability and willingness to report to work during public health emergencies.	*Disaster medicine and public health preparedness*	To assess the ability and willingness of health care workers to report to work during a pandemic influenza and a severe earthquake and to identify barriers and strategies that would help them report to work.
Stergiou, D. P.; Farmaki, A. [133]	2021	Ability and willingness to work during COVID-19 pandemic:Perspectives of front-line hotel employees.	*International Journal of Hospitality Management*	To identify factors that may influence their ability and willingness to report to work.
Sultan, M. et al. [155]	2020	Emergency Healthcare Providers' Perceptions of Preparedness and Willingness to Work during Disasters and Public Health Emergencies.	*Healthcare*	To evaluates the perceptions of preparedness and willingness to work during disasters and public health emergencies.
Syrett, J. I. et al. [54]	2007	Will emergency health care providers respond to mass casualty incidents?	*Prehospital emergency care*	To work during a mass casualty emergency regardless of family duties, type of incident, or availability of treatment.
Tippett, V. et al. [114]	2020	Anticipated Behaviors of Emergency Prehospital Medical Care Providers during an Influenza Pandemic.	*Prehospital and Disaster Medicine*	To investigate the association between knowledge and attitudes regarding avian influenza on likely behavioral responses of Australian emergency prehospital medical care providers in pandemic conditions.
Tong, L. K. et al. [73]	2021	Nurses Who Are More Willing to Participate in the Fight against COVID-19: Evidence from China.	*International journal of environmental research and public health*	To assess the willingness of nurses to participate in the fight against COVID-19 in China and to identify factors associated therewith.
Trigueiro, R. L. et al. [85]	2020	COVID-19 pandemic: report on the use of auriculotherapy to optimize emergency workers' health.	*Revista brasileira de enfermagem*	To report the use of auriculotherapy to optimize emergency workers' health during the COVID-19 pandemic.
Upadhyaya, D. P. et al. [84]	2020	Frontline Healthcare Workers' Knowledge and Perception of COVID-19, and Willingness to	*Healthcare*	To investigate the contextual factors associated with the knowledge, perceptions, and the willingness of frontline healthcare workers (FHWs) to work during the COVID-19 pandemic in Nepal.

		Work during the Pandemic in Nepal		
von Gottberg, C.; Krumm, S.; Porzsolt, F.; Kilian, R. [136]	2016	The analysis of factors affecting municipal employees' willingness to report to work during an influenza pandemic by means of the extended parallel process model (EPPM).	*BMC public health*	To understand the factors that influence municipal employees' willingness to come to work during a pandemic is therefore a basic requirement for adequate public health preparedness.
Wilke, J. et al. [134]	2020	Restrictercise! Preferences Regarding Digital Home Training Programs during Confinements Associated with the COVID-19 Pandemic.	*International journal of environmental research and public health*	To identify preferences regarding digital home training programs during confinements associated with the Covid-19 pandemic.
Wong, E. L. et al. [50]	2012	Healthcare workers' duty concerns of working in the isolation ward during the novel H1N1 pandemic.	*Journal of clinical nursing*	To explore healthcare workers' duty concerns in the isolation wards during the novel H1N1 pandemic.
Woyessa AH. et al. [107]	2021	Predictors of Health-Care Workers' Unwillingness to Continue Working During the Peak of COVID-19 in Western Ethiopia: An Extended Parallel-Process Model Study	*Risk Manag Healthc Policy*	To work in disasters is context-specific and corresponds to the nature, magnitude, and threats posed by a particular public health emergency.
Wu, B. et al. [58]	2020	Factors associated with nurses' willingness to participate in care of patients with COVID-19: A survey in China	*Journal of nursing management*	To assess nurses' willingness to participate in care of patients with coronavirus disease 2019 (COVID-19) in China and to identify its associated factors.
Xu, X. et al. [106]	2021	Suicidal and self-harm ideation among Chinese hospital staff during the COVID-19 pandemic: Prevalence and correlates.	*Psychiatry research*	To detect the prevalence of suicidal and self-harm ideation (SSI) and its related risk factors among hospital staff during the COVID-19 pandemic.

Young, C. F.; Persell, D. J. [159]	2004	Biological, chemical, and nuclear terrorism readiness: major concerns and preparedness of future nurses.	*Disaster management & response: DMR*	To identify student nurses' major concerns in relation to working with victims of terrorism.
Zewudie, A. et al. [37]	2021	Healthcare Professionals' Willingness and Preparedness to Work During COVID-19 in Selected Hospitals of Southwest Ethiopia.	*Risk management and healthcare* policy	To Identify barriers of willingness to work during epidemics outbreak and preparedness of healthcare professionals.
Zhang, K. et al. [97]	2021	Psychological Burden and Experiences Following Exposure to COVID-19: A Qualitative and Quantitative Study of Chinese Medical Student Volunteers.	*International journal of environmental research and public health*	To conduct a timely assessment of the psychological burden and experience on medical student volunteers during the COVID-19 pandemic.
